# Modulation of Cell Adhesion and Migration by the Histone Methyltransferase Subunit mDpy-30 and Its Interacting Proteins

**DOI:** 10.1371/journal.pone.0011771

**Published:** 2010-07-23

**Authors:** Bin Xia, Alexandra Joubert, Benjamin Groves, Kevin Vo, Davin Ashraf, Derek Djavaherian, Jason Awe, Ying Xiong, Jacqueline Cherfils, Dzwokai Ma

**Affiliations:** 1 Department of Pediatrics, Sichuan University, West China Second University Hospital, Chengdu, China; 2 Department of Molecular, Cellular, and Developmental Biology, University of California Santa Barbara, Santa Barbara, California, United States of America; 3 Laboratoire d'Enzymologie et Biochimie Structurales, CNRS, Gif sur Yvette, France; 4 Neuroscience Research Institute, University of California Santa Barbara, Santa Barbara, California, United States of America; University of Nebraska Medical Center, United States of America

## Abstract

We have previously shown that a subset of mDpy-30, an accessory subunit of the nuclear histone H3 lysine 4 methyltransferase (H3K4MT) complex, also localizes at the trans-Golgi network (TGN), where its recruitment is mediated by the TGN-localized ARF guanine nucleotide exchange factor (ArfGEF) BIG1. Depletion of mDpy-30 inhibits the endosome-to-TGN transport of internalized CIMPR receptors and concurrently promotes their accumulation at the cell protrusion. These observations suggest mDpy-30 may play a novel role at the crossroads of endosomal trafficking, nuclear transcription and adhesion/migration. Here we provide novel mechanistic and functional insight into this association. First, we demonstrate a direct interaction between mDpy-30 and BIG1 and locate the binding region in the N-terminus of BIG1. Second, we provide evidence that the depletion or overexpression of mDpy-30 enhances or inhibits cellular adhesion/migration of glioma cells *in vitro*, respectively. A similar increase in cell adhesion/migration is observed in cells with reduced levels of BIG1 or other H3K4MT subunits. Third, knockdown of mDpy-30, BIG1, or the RbBP5 H3K4MT subunit increases the targeting of β1 integrin to cell protrusions, and suppression of H3K4MT activity by depleting mDpy-30 or RbBP5 leads to increased protein and mRNA levels of β1 integrin. Moreover, stimulation of cell adhesion/migration via mDpy-30 knockdown is abolished after treating cells with a function-blocking antibody to β1 integrin. Taken together, these data indicate that mDpy-30 and its interacting proteins function as a novel class of cellular adhesion/migration modulators partially by affecting the subcellular distribution of endosomal compartments as well as the expression of key adhesion/migration proteins such as β1 integrin.

## Introduction

Covalent modifications of histones are crucial for the modulation of chromatin structure and function. One form of histone modification is the methylation of histone H3 lysine 4 residues (H3K4), which is enriched near the transcription start sites of actively transcribed genes in both yeast and mammals (1). H3K4 methyltransferases (H3K4MT) methylate H3K4, and the majority of mammalian H3K4MTs consist of one catalytic subunit and several common accessory subunits, including Ash2L, RbBP5, WDR5, and mDpy-30 (mammalian ortholog of Dpy-30 protein) [1]. Current evidence suggests that these accessory subunits play an important role in the specificity and activity of the catalytic subunits [2]. Whereas a plethora of information has been obtained regarding the machinery that modifies H3K4 methylation [3], little is known about the physiological consequences of this modification.

Our group has recently shown that a pool of mDpy-30, a protein originally identified as an essential component of the dosage compensation machinery in *C. elegans* [4], is recruited to the TGN via its interaction with BIG1 [5], a TGN-localized ARF GEF [6], [7], [8]. We have previously shown that depletion of mDpy-30 with siRNA slows the endosome-to-TGN transport of internalized cation independent mannose-6-phosphate receptor (CIMPR) and causes these receptors to accumulate near cell protrusions without affecting the distributions of TGN46 or TfnR. Suppression of either Ash2L or RbBP5 causes a similar enrichment of CIMPR at cell protrusions. Moreover, Rab4 and Rab11, two GTPases which regulate endosomal recycling, are also enriched at the protrusions of mDpy-30 knockdown cells [5]. Thus, H3K4MT subunits likely regulate the endosomal recycling of specific cargo proteins to cell protrusions. Given the intimate link between cell protrusions and cell motility, the above observations prompted us to examine the role of mDpy-30 and its interacting proteins in cell adhesion/migration.

## Results and Discussion

For the characterization of siRNAs and lentiviral expression constructs, see the supplementary information (Fig. S1, S2, S3).

### Characterizations of the interaction between BIG1 and mDpy-30

**Figure 1:**
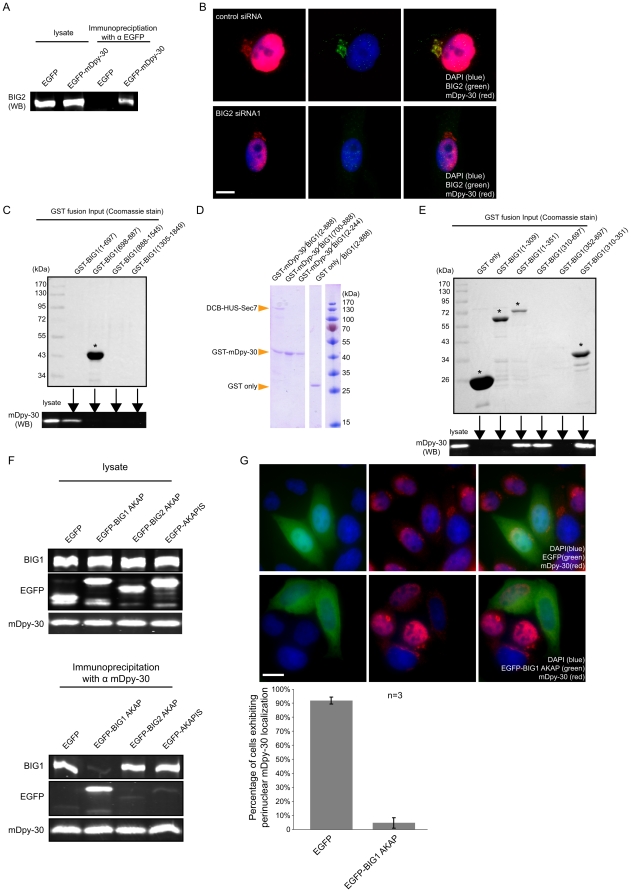
Characterization of the interaction between mDpy-30 and two ARF1 GEFs (BIG1 and BIG2). (A) The ability of EGFP and EGFP-mDpy-30 to co-immunoprecipitate BIG2 was examined in HeLa cells by western blotting. (B) The effect of BIG2 knockdown on mDpy-30 perinuclear localization was assayed by immunofluorescence in HeLa cells. (C) GST fusions of various BIG1 domains were purified and their abilities to pull down mDpy-30 from HeLa cell lysates were investigated by western blotting. Crude cell lysate was included at 1/30 the level as a reference in the western blot. The input levels of GST fusions were assessed with Coomassie staining. (D) GST pull-down analyses of the direct binding between recombinant mDpy-30 and the N-terminus of BIG1. (E) Pull-down efficiencies of mDpy-30 with the GST fusions of additional BIG1 N-terminal fragments, performed as in (C). In (C) and (E) Full-length GST-fusion proteins are indicated by asterisks. Note that the yield of some GST fusions is too low to be visualized (those lanes without asterisks). (F) Co-immunoprecipitation analyses of mDpy-30 and BIG1 in HeLa cells expressing EGFP alone or EGFP fusions of AKAP sequences from BIG1, BIG2 or AKAPIS. mDpy-30 was immunopreciptated and the levels of associated BIG1 and the various EGFP-AKAP fusions were assayed by western blotting (right panels). The blots on the left panels correspond to the crude lysates. (G) The perinuclear localization of mDpy-30 was observed using immunofluorescence in the presence of either the EGFP fusion of the BIG1 AKAP domain or EGFP alone. Each experiment was performed three times (n = 3) and more than 250 cells were scored in each condition (representative images shown). Error bars: standard deviation; scale bars: 10 µm.

We have shown that BIG1 recruits mDpy-30 to the TGN [5]. BIG2, a homolog of BIG1, is another ARFGEF found at the TGN [9]. Given the similarity between these two proteins, we explored whether mDpy-30 interacts with BIG2 and if so, whether this interaction also contributes to the TGN localization of mDpy-30. As shown in Fig. 1A, BIG2 can be co-immunoprecipitated with EGFP-mDpy-30, indicating that the two proteins can be found in a complex. However, unlike the knockdown of BIG1, which leads to a great reduction of TGN mDpy-30 [5], depletion of BIG2 had no appreciable effect on the mDpy-30 perinuclear localization (Fig. 1B) Given this data, we decided to focus on the mDpy-30/BIG1 interaction and conducted a series of GST pull-down assays. We produced several BIG1 constructs fused to GST and used them to pull-down mDpy-30 from HeLa cell extracts. We found that mDpy-30 was pulled down by a BIG1 construct encompassing its N-terminal dimerization DCB/HUS domains (residues 1-697), but not by its catalytic Sec7 domain (residues 698–887) (Fig. 1C). Due to the low yields of the BIG1 C-terminal fusion proteins (residues 888–1545; residues 1305–1849) (Fig. 1C), no conclusion could be made about the ability of the C-terminal region of BIG1 to interact with mDpy-30. To establish whether a direct interaction occurs between the N-terminus of BIG1 and mDpy-30, we used purified recombinant BIG1 N-terminal constructs and GST-tagged mDpy-30 *in vitro*. Consistent with the above analysis, we detected direct binding between mDpy-30 and the BIG1 N-terminal fragment spanning the DCB, HUS and Sec7 domains (residues 2–888)), but not between mDpy-30 and the Sec7 domain (residues 700–888) or DCB domain (residues 2–244) alone (Fig. 1D).

The crystal structure of the C-terminus of mDpy-30 shows that it forms a dimer with a topology reminiscent of the dimerization and docking (DD) domains of the regulatory subunits of PKA [10]. The DD domains bind to proteins that contain an A kinase anchoring protein (AKAP) sequence, which is a 12–14 helical peptide with a ΦΦxxΦΦxxΦΦxxΦΦ signature, where Φ is a hydrophobic residue (mostly A, I, L or V) [11], [12]. Interestingly, three AKAP sequences in the N-terminus region of BIG2 [13], and one in the N-terminus of BIG1 (IVQNIVEEMVNIVV,) (Ishizaki et al., 2006) have been predicted. In order to examine whether this AKAP sequence mediates the mDpy-30/BIG1 interaction, we performed pull-down analysis with additional GST-fused N-terminal BIG1 constructs. We found that only those BIG1 constructs (residues 1–351, 310–697, and 310–351) that encompass the putative AKAP sequence (residues 328–341) were able to pull down mDpy-30 (Fig. 1E). Judging from the fact that GST-DCB/AKAP (residues 1–351) or GST-AKAP/HUS (residues 310–697), both expressed at lower levels compared to GST-AKAP (residues 310–351), were able to pull down similar amounts of mDpy-30 as GST-AKAP, it is probable that the AKAP-mediated interaction between BIG1 and mDpy-30 is enhanced by the presence of either the DCB or HUS domains.

To further confirm the essential role of AKAP sequence in the binding between BIG1 and mDpy-30, we investigated whether the expression of a GFP-AKAP affected the BIG1/mDpy-30 association. Indeed, the expression of this fusion in HeLa cells prevented co-immunoprecipitation of BIG1 with mDpy-30 (Fig. 1F). As a control, the GFP fusions of either a BIG2 AKAP sequence or AKAPIS, a DD domain binding peptide [14], both failed to interfere with the BIG1/mDpy-30 interaction (Fig. 1F). Consistent with this result, endogenous mDpy-30 perinuclear localization was lost or greatly reduced only when the BIG1 AKAP sequence is expressed (Fig. 1G), but not when either the BIG2 AKAP sequence, or AKAPIS were present (data not depicted). The loss of mDpy-30 perinuclear staining was not due to the disruption of the Golgi apparatus, as determined by β-GalT1 staining (data not depected).

We conclude from this ensemble of experiments that the N-terminus of BIG1 binds directly to mDpy-30. The AKAP sequence of BIG1 is critical for this interaction, but the association between BIG1 and mDpy-30 is likely further strengthened by the presence of an integral DCB/HUS dimerization region of BIG1 that encompasses the putative AKAP sequence. This conclusion is consistent with the observation, based on the published crystal structure [10], that the surface topology of the C-terminus of mDpy-30 may not readily accommodate an AKAP helix alone. In light of the recent studies suggesting that BIG1 and BIG2 can form a heteromeric complex [15], it seems likely that the co-immunoprecipitation of BIG2 by mDpy-30 is the result of an indirect interaction.

### Enrichment of endogeneous Rab11 and Rab4 at the protrusions of mDpy-30 knockdown cells

**Figure 2:**
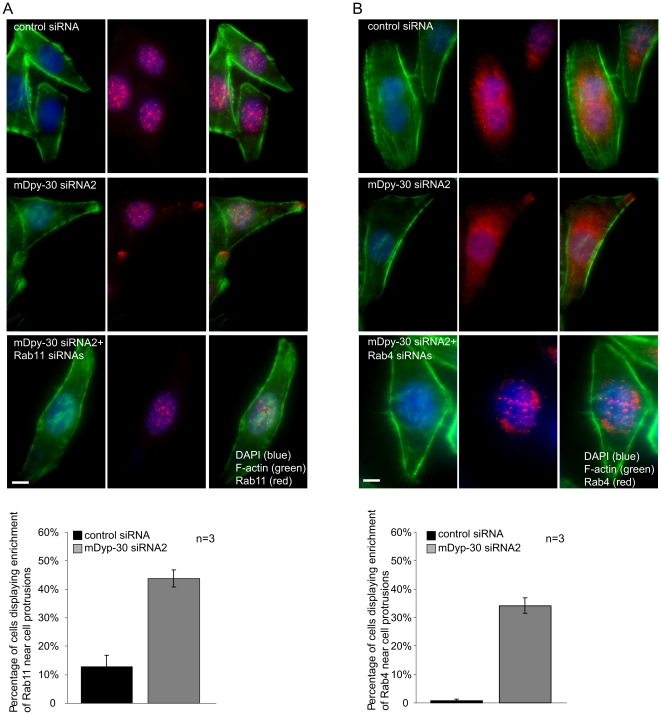
Depletion of mDpy-30 on the protrusion targeting of endogenous Rab4 and Rab11. (A–B) HeLa Cells were transfected with 20 nM non-targeting control siRNA or mDpy-30 siRNA for 48 hr before analysis. The percentage of cells displaying enriched staining of endogenous Rab4 or Rab11 at cell protrusions was determined based on its proximity to peripheral F-actin revealed by phalloidin staining. Whereas both the anti-Rab4 and anti-Rab11 antibodies produced non-specifc nuclear staining, the specificity of the protrusion staining was verified using siRNAs against Rab4 (Rab4A and B, two isoforms present in HeLa) and Rab11 (Rab11A and B, two isoforms present in HeLa). Each experiment was performed three times (n = 3) and more than 250 cells were scored in each condition (representative images shown) Error bars: standard deviation; scale bars: 10 µm.

Using the EGFP fusions of various Rab proteins, we previously reported that Rab11 and Rab4 become enriched near cell protrusions after the depletion of mDpy-30 [5]. To explore whether endogenous Rab11 and Rab4 behave in a similar way, we examined the protrusion targeting of both proteins in cells depleted of mDpy-30 using commercial antibodies. Although both of the commercial antibodies display non-specific background staining (particularly nuclear), they also exhibit enhanced protrusion staining in mDpy-30 knockdown cells, that is lost after siRNA treatment (Figs. 2A and 2B, respectively). This result indicates that endogenous Rab4 and Rab11 are also enriched at the cell protrusions as a result of mDpy-30 knockdown.

### Colocalization of internalized CIMPR with EGFP-Rab4 compartments near the protrusions of mDpy-30 knockdown cells

**Figure 3:**
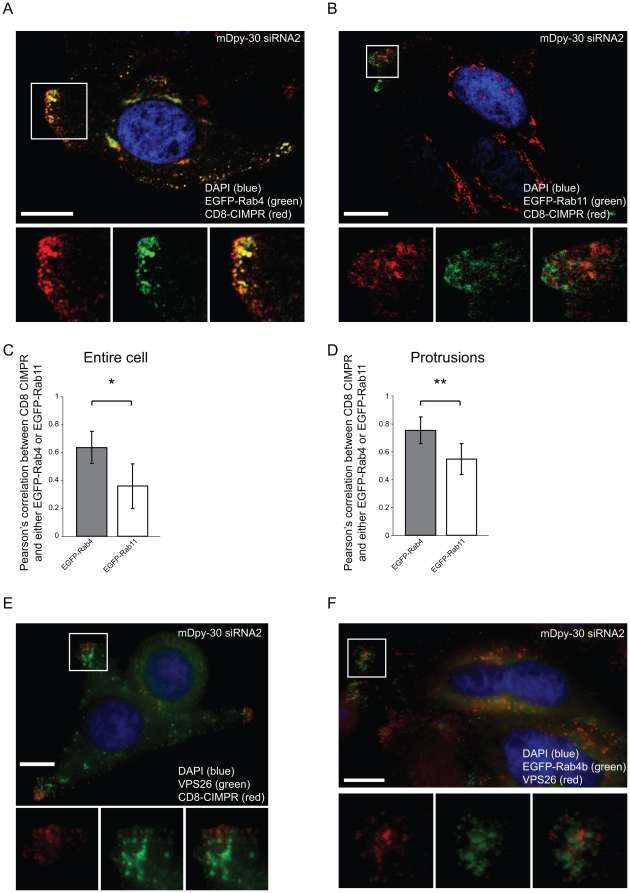
Colocalization analyses between internalized CD8-CIMPR and EGFP-Rab4b or EGFP-Rab11 recycling GTPases in mDpy-30 knockdown cells. HeLa cells were transfected with 20 nM siRNAs for 48 hr and/or cDNA for 24 hr before analysis. (A–B) Colocalization analyses of internalized CD8-CIMPR and EGFP-Rab4b (A) or EGFP-Rab11 (B) in HeLa CD8-CIMPR stable cells treated with an mDpy-30 siRNA. A monoclonal anti-CD8 antibody was added to the culture media (37°C, 45 min) to allow the labeling and internalization of surface CD8-CIMPR [5], and the localizations of internalized receptors and both Rab GTPases were examined using a confocal microscope. (C–D) Quantification of colocalization between internalized CD8-CIMPR and either EGFP-Rab4b or EGFP-Rab11 in the entire cell (C) or the protrusions (D) of HeLa CD8-CIMPR cells depleted of mDpy-30. The Pearson's correlations between the signals in single 0.5 µm Z-sections of confocal images were calculated. At least 20 cells were measured for each condition. (E–F) Colocalization analyses between VPS26 and internalized CD8-CIMPR (F) or EGFP-Rab4b (F) using a conventional microscope. Error bars: standard deviation; scale bars: 10 µm. Asterisks indicate a p-value of ≤0.05 (*) or ≤0.01(**) as determined by Student's T-test.

To test whether mDpy-30 depletion indeed causes CIMPR to be targeted to a recycling compartment at the protrusions, we conducted a colocalization study between internalized CD8-CIMPR and Rab4 or Rab11 in HeLa cells depleted of mDpy-30. Due to the background associated with the Rab antibodies as well as their overall weak staining, we instead expressed the EGFP fusion of Rab4b and Rab11. As shown in Figs. 3A and 3B, internalized CD8-CIMPR appears to colocalize more with EGFP-Rab4b than with EGFP-Rab11. This was verified using a statistical colocalization analysis of confocal images (Fig. 3C and 3D). Furthermore, both CD8-CIMPR and EGFP-Rab4b present at the protrusions were largely segregated from VPS26 (Figs. 3E and 3F) and SNX2 (data not depicted), two subunits of the retromer complex known to mediate the endosome-to-TGN transport of CIMPR [16]. Our data suggests that a pool of internalized CD8-CIMPR proteins accumulate at the protrusions of mDpy-30 knockdown cells where they reside in EGFP-Rab4b positive compartments.

### Identification of β1 integrin as another mDpy-30-dependent cargo protein

**Figure 4:**
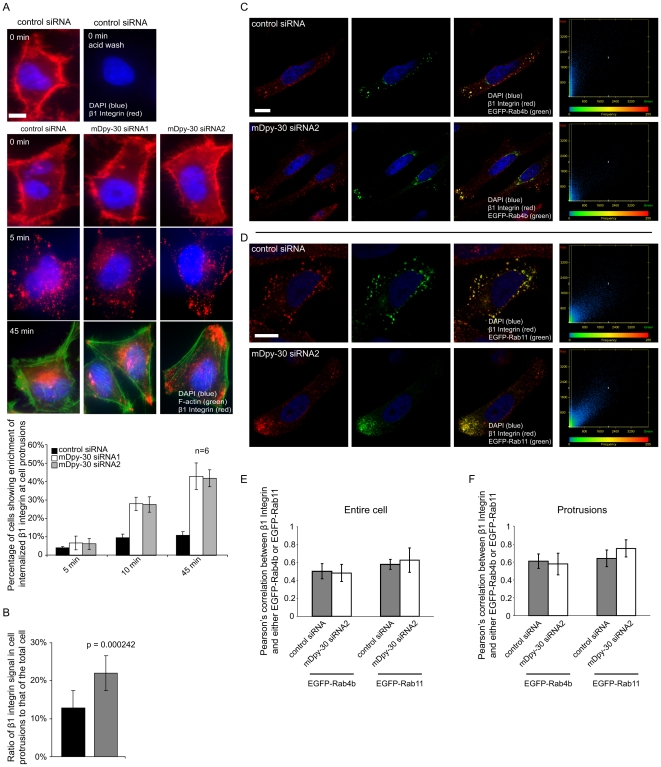
Colocalization analyses between internalized β1 integrin and EGFP-Rab4b or EGFP-Rab11 recycling GTPases in mDpy-30 knockdown cells. HeLa cells were transfected with 20 nM siRNAs for 48 hr and/or cDNA for 24 hr before analysis. (A) Impact of depleting mDpy-30 on the endosomal trafficking of β1 integrin. An anti-β1 integrin antibody was employed to label surface β1 integrin (0°C, 1 hr). After labeling, cells were returned to 37°C for various periods of time before imaging analysis. A brief acid wash was conducted as described in “Materials and Methods” before fixation to remove surface-bound antibody and permit the better visualization of internalized β1 integrin. The enrichment of internalized β1 integrin near cell protrusions was scored as described in Fig. 2. The experiments were performed six times (n = 6) with more than 500 cells scored in each experiment. (B) The fraction of total internalized β1 integrin that appears at cell protrusions was determined from confocal images of cells treated with either a non-targeting control siRNA or mDpy-30 siRNA. Only cells exhibiting internalized β1 integrin at cell protrusions were assayed from both conditions. At least 50 cells from each condition were used for the quantification. The average number of protrusions per cell did not vary significantly in the two conditions (1.2 for control and 1.3 for mDpy-30 siRNA); p-value determined by Student's T-test (C–D) Colocalization analyses of internalized β1 integrin and either EGFP-Rab4b (C) or EGFP-Rab11 (D) using confocal microscopy. An anti-β1 integrin antibody was added to the culture medium (37°C, 45 min) to allow the labeling and internalization of β1 integrin. The graphs at the right indicate the signal intensity of each pixel measured in the single representative Z-section images that appear to the left. The X-axis is the intensity of EGFP-Rab4b or EGFP-Rab11 signal and the Y-axis corresponds to that of internalized β1 integrin. The color on the graph corresponds the relative frequency with which a specific combination of X and Y values is found in all the pixels analyzed, with blue indicating lower frequencies and red higher frequencies. (E–F) Quantification of colocalization between internalized β1 integrin and either EGFP-Rab4b or EGFP-Rab11 in the entire cell (E) or the protrusions (F) of cells depleted of mDpy-30. The Pearson's correlations were determined as described in Fig. 3C and 3D. Error bars: standard deviation; scale bars: 10 µm.

Since the formation of cell protrusions is the first step towards migration, we wondered whether mDpy-30 also regulates the subcellular distribution of integrins, a protein family that plays a key role in cell adhesion/migration. To test this possibility, we examined the impact of depleting mDpy-30 on the trafficking of β1 integrin, which is widely expressed and has the capacity to control cell adhesion/migration on many extracellular matrix (ECM) substrates by forming a functional heterodimer with multiple α integrins [17]. Similar to what was previously observed for CD8-CIMPR [5], a time-course study of β1 integrin internalization indicated that mDpy-30 knockdown caused internalized β1 integrin to be redirected to cell protrusions (Fig. 4A). Moreover, quantitative analysis of confocal images confirmed that mDpy-30 knockdown resulted in an increased protrusion-to-total ratio of internalized β1 integrin without affecting the average number of protrusions within a cell (Fig. 4B).

There are at least two possible explanations for the above observations. The first is that the depletion of mDpy-30 causes a change in the trafficking of β1 integrin, either through redirection to a new compartment, or through redistribution along the normal pathway. The second is that mDpy-30 knockdown changes the location of the trafficking compartments themselves, without altering β1 integrin trafficking. In order to distinguish between these two possibilities we performed a quantitative colocalization analysis between internalized β1 integrin and EGFP-Rab4b or EGFP-Rab11. This investigation revealed that internalized β1 integrin displayed significant colocalization with EGFP-Rab11, and to a slightly lesser extent with EGFP-Rab4b, both of which was not affected by mDpy-30 knockdown (Fig. 4C–F). When combined with our studies showing that exogenously [5] and endogenously (Fig. 2) expressed Rab4b and Rab11 become enriched in the protrusions of cells depleted of mDpy-30, these data suggest that instead of changing the trafficking of β1 integrin, mDpy-30 knockdown leads to the spatial redistribution of the trafficking compartments themselves.

### mDpy-30-mediated inhibition of cell adhesion and migration

**Figure 5:**
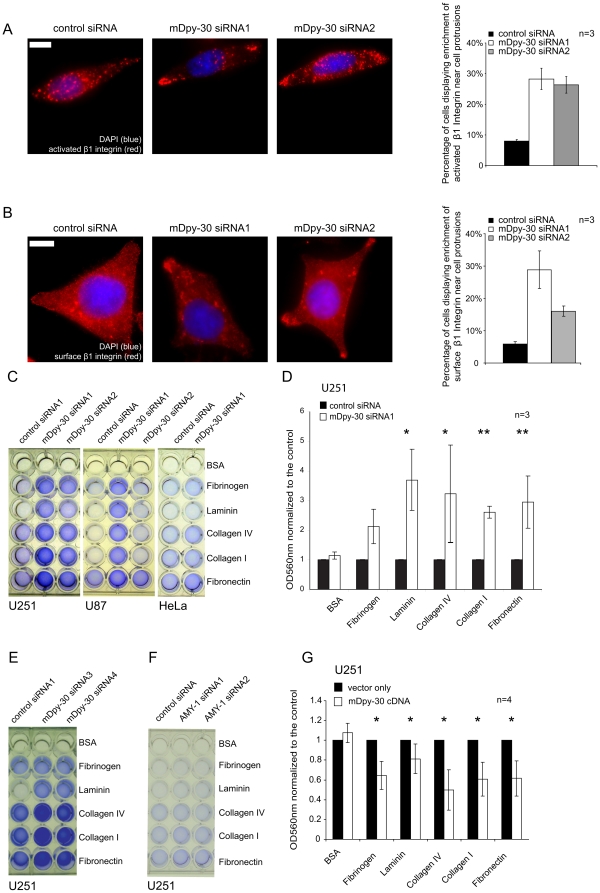
Impact of mDpy-30 knockdown and overexpression on cell adhesion. Cells were transfected with 20 nM siRNAs for 48 hr or infected with mDpy-30-expressing lentiviral particles for 6 days before analysis. (A) HeLa cells were treated with a non-targeting control siRNA, mDpy-30 siRNA1 or mDpy-30 siRNA2. The fraction of cells displaying enrichment of internalized activated β1 integrin at cell protrusions was determined. (B) HeLa cells were treated as above. The surface β1 integrin was visualized using antibody staining under non-permeabilizing conditions. In (A) and (B) each experiment was performed three times (n = 3) and at least 50 cells were scored in each condition (representative images shown). (C) Influence of mDpy-30 knockdown on the adhesion of U251, U87 and HeLa cells assessed with an ECM array. (D) The adhesion of U251 cells was quantified using OD_560 nm_ as described in “Materials and Methods” and normalized to the control. Only the result from one siRNA (siRNA1) was shown here but a similar adhesion enhancement (albeit with a slightly reduced effect) was obtained using the other siRNA (siRNA2). (E) Effect of mDpy-30 suppression on U251 adhesion using a different pair of mDpy-30 siRNAs which contain modifications to minimize non-specific target effects. (F) Impact of AMY-1 depletion on U251 adhesion. The experiment was repeated once and a similar observation was made. (G) Influence of mDpy-30 overexpression on U251 adhesion. Variable “n” on each bar graph represents the number of experiments performed. Error bars: standard deviation; scale bars: 10 µm. Single and double asterisks indicate a p-value (determined by a paired Student's T-test) of ≤0.05 and ≤0.01, respectively.

We next assessed whether the population of internalized β1 integrin which accumulated at the protrusions of mDpy-30 knockdown cells represented activated β1 integrin, a form of β1 integrin with higher ligand binding activity [18], [19]. Indeed, reduction of mDpy-30 also led to an increase in the number of cells with activated β1 integrin near cell protrusions (Fig. 5A). Surface labeling further showed that there was a corresponding increase of β1 integrin at the plasma membrane of cell protrusions after mDpy-30 knockdown (Fig. 5B), suggesting that internalized β1 integrin likely recycled to the surface via the local recycling compartments at the protrusions.

Polarized trafficking of integrins has been known to mediate cell adhesion/migration [20], [21], thus the above observations prompted us to investigate the role of mDpy-30 in these processes. We first utilized an ECM array to determine how knockdown of mDpy-30 affected the adhesion of HeLa and two human glioma cell lines, U251 and U87 (ATCC, Rockville, MD, USA). Glioma cells are well known for being characteristically invasive and migratory, and the importance of β1 integrin in controlling glioma invasion/migration has been documented in many previous studies [22]. As in the case of HeLa cells, mDpy-30 depletion resulted in the enrichment of internalized β1 integrin at the protrusions of U251 (Fig. S4) and U87 (data not depicted) cells. The results in Figs. 5C and 5D show that even though mDpy-30 siRNA treatment did not change the non-specific cell adhesion on BSA, it significantly enhanced the adhesion of U251 and U87 on all ECM substrates tested (with the exception of Fibrinogen). A moderate increase in adhesion was also observed for HeLa cells. Because U251 adhesion exhibited a higher sensitivity to the mDpy-30 siRNAs, we performed a time-course study of adhesion (Fig. S5) using this cell line to determine the optimal time point (i.e. 40 min after seeding) for subsequent adhesion experiments. Further, we carried out the following three experiments to exclude potential non-specific siRNA effects. First, we repeated the experiment using a different set of mDpy-30 siRNAs, which target different sequences of mDpy-30 mRNA and contain a unique chemical modification pattern to reduce off-target effects (ON-TARGETplus siRNA, Dharmacon). A similar enhancement of adhesion was noted in cells treated with these siRNAs (Fig. 5E). Second, treating U251 cells with siRNAs against AMY-1, a BIG2-interacting protein [23], had little influence on cell adhesion (Fig. 5F). Third, and most importantly, overexpression of mDpy-30 decreased the level of adhesion, opposing the phenotype displayed when mDpy-30 is depleted in cells (Fig. 5G).

**Figure 6:**
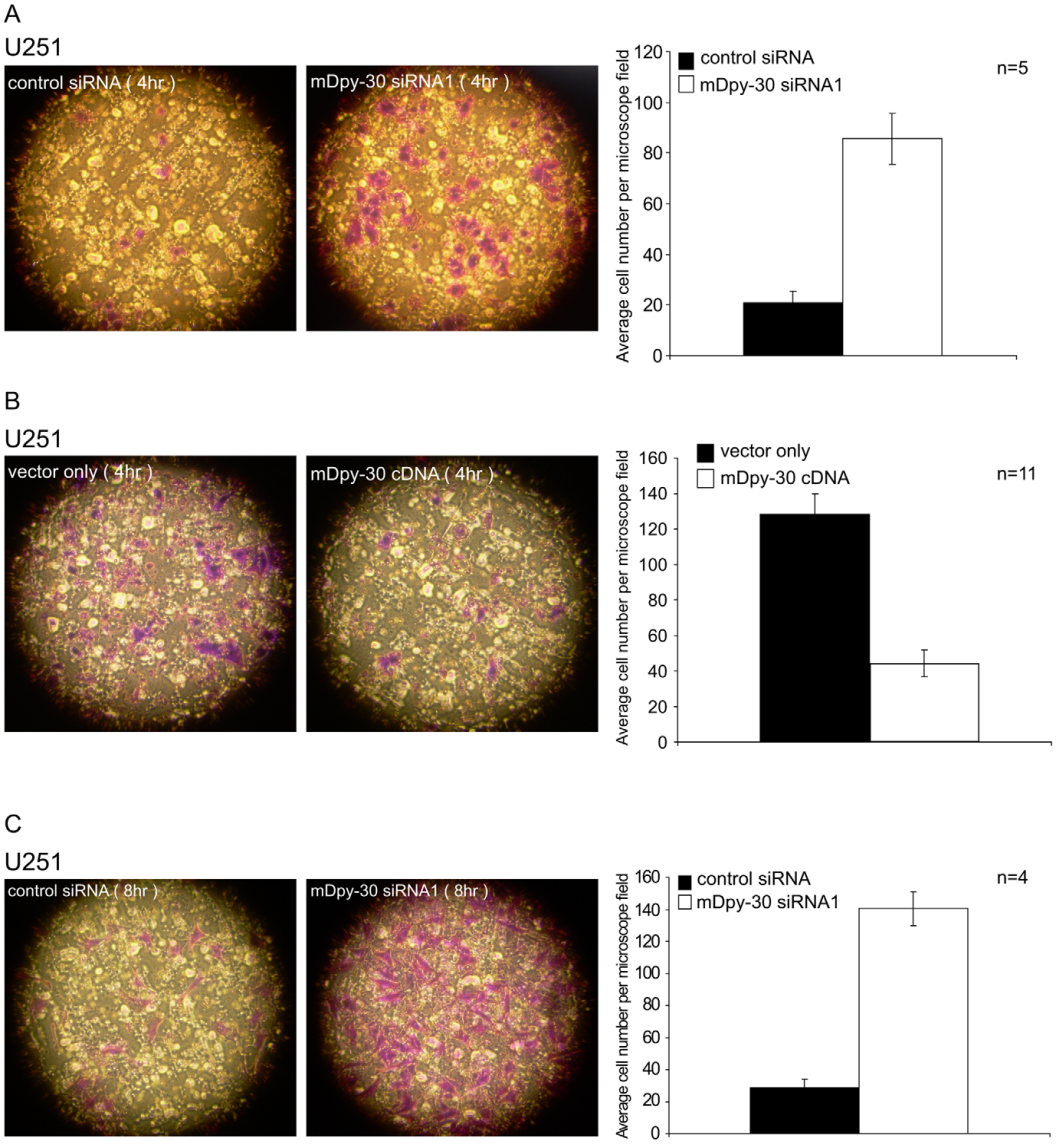
Impact of mDpy-30 knockdown and overexpression on cell migration. U251 cells were treated as described in Fig. 5. Effects of mDpy-30 knockdown (A) and overexpression (B) on U251 migration assayed with a transwell migration chamber. (C) Effect of mDpy-30 knockdown on U251 haptotaxis assayed with a transwell migration chamber containing a Collagen I coated membrane. The migratory cells were stained at 4 hr after seeding, and counted under a microscope. Representative fields are shown. The experiment was repeated once and a similar result was obtained. Variable “n” on each bar graph represents the number of microscopic fields examined (for migration). Error bars: standard deviation.

Since productive cell migration requires an optimal level of adhesion, these observations indicate that mDpy-30 plays either an inhibitory or stimulatory role in the migratory pathway. To address this question, we investigated how different levels of mDpy-30 expression impacted the ability of U251 cells to migrate across a polycarbonate membrane insert towards chemoattractants present in serum. Based on the result of a pilot time-course experiment (Fig. S6), we chose to conduct our migration study 4 hr after seeding. Our result revealed that suppression (Fig. 6A) of mDpy-30 enhanced the migration of U251 while its overexpression (Fig. 6B) decreased it. Moreover, mDpy-30 knockdown also resulted in increased levels of haptotaxis (Fig. 6C). When combined, our data demonstrates that mDpy-30 plays an inhibitory role in cell adhesion/migration.

### Roles of mDpy-30-interacting proteins, including BIG1 and other H3K4MT subunits, in subcellular distribution of CIMPR and β1 integrin, as well as in cell adhesion/migration

**Figure 7:**
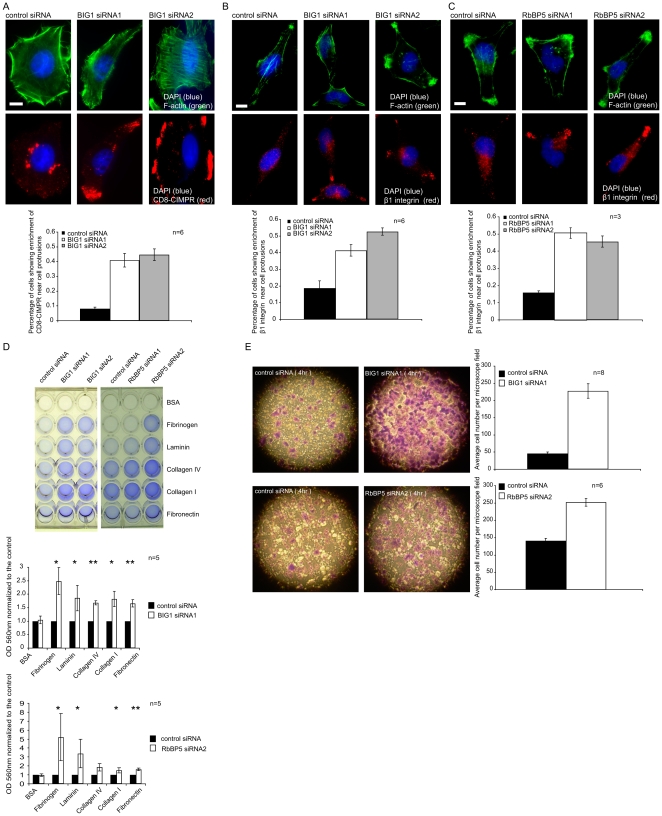
Effects of BIG1 or H3K4MT knockdown on the protrusion targeting of CD8-CIMPR and β1 integrin as well as cell adhesion/migration. Cells were transfected with 20 nM siRNAs for 48 hr before analysis. (A-B) Impact of BIG1 knockdown on the protrusion targeting of internalized CD8-CIMPR in HeLa stable cells (A), or internalized β1 integrin in HeLa cells (B). Internalization of CD8-CIMPR and β1 integrin was conducted as described in Figs. 3 and 4, respectively. (C) Influence of RbBP5 knockdown on the protrusion targeting of internalized β1 integrin in HeLa cells. (D–E) Effects of BIG1 and RbBP5 siRNAs on the adhesion (D) and migration (E) of U251 cells. Cell adhesion and migration assays were performed as described in Figs. 5 and 6. HeLa and U251 cells were used for imaging and adhesion/migration studies, respectively, because the former has a flat morphology while the latter displays a greater sensitivity to the effect of mDpy-30 knockdown on adhesion/migration. However, CIMPR and β1 integrin are similarly enriched in U251 cells depleted of BIG1 or RbBP5 (data not depicted). Variable “n” on the bar graphs represents the number of experiments performed (for imaging and adhesion) or the number of microscopic fields examined (for migration). Error bars: standard deviation; scale bars: 10 µm. Single and double asterisks indicate a p-value (determined by a paired Student's T-test) of ≤0.05 and ≤0.01, respectively.

To gain insight into which subset of mDpy-30 (nuclear or cytoplasmic) modulates cell trafficking/adhesion/migration, we conducted expression studies on two of its differently localized interacting proteins. In one experiment, we assessed the role of BIG1, which recruits mDpy-30 to the TGN. Mimicking our observations from when mDpy-30 siRNAs were used, knockdown of BIG1 caused an accumulation of internalized CD8-CIMPR (Fig. 7A) and β1 integrin (Fig. 7B) at the protrusions of both HeLa and U251 (data not shown) cells. We also tested the effects of RbBP5 knockdown since it is known to to form a complex with mDpy-30 in the nucleus as part of the H3K4MT. Similar to our previous observation that RbBP5 depletion led to the enrichment of CD8-CIMPR at cell protrusions [5], siRNAs against RbBP5 increased the targeting of internalized β1 integrin to these sites (Fig. 7C). Finally, suppression of either BIG1 or RbBP5 both enhanced the adhesion (Fig. 7D) and migration (Fig. 7E) of U251 cells. Thus, mDpy-30 and its interacting proteins, within both nuclear and cytoplasmic pools, function as negative modulators in the cell adhesion/migration pathway.

### Regulation of β1 integrin gene expression by mDpy-30 and other H3K4MT subunits

**Figure 8:**
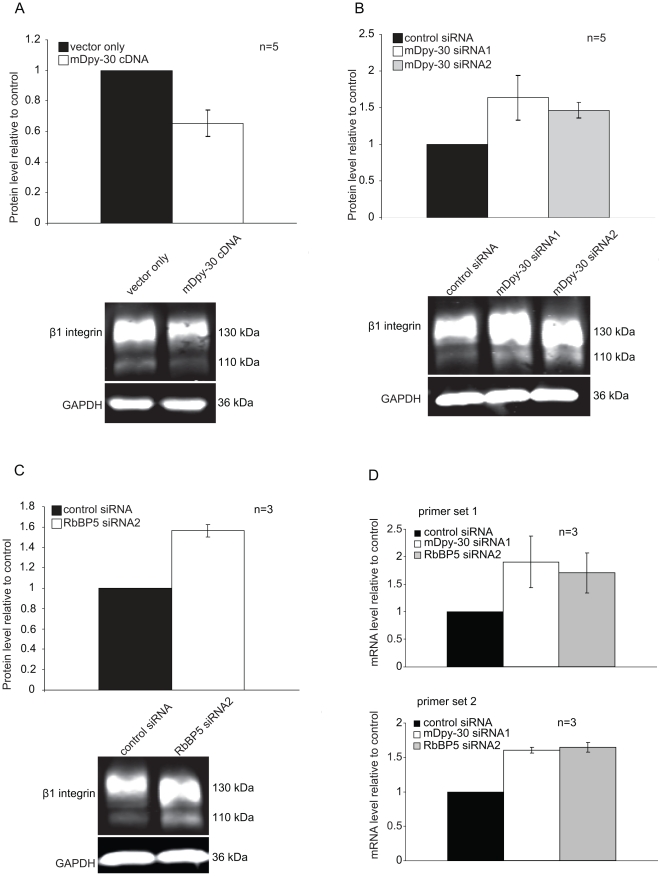
Regulation of protein and mRNA levels of β1 integrin mediated by mDpy-30 and RbBP5. U251 cells were transfected with 20 nM siRNAs for 48 hr or infected with mDpy-30-expressing lentiviral particles for 6 days before analysis. For western blot analyses, an equal amount of the total protein was loaded into each lane and GAPDH was used as a loading control. The band intensity was quantified using the Odyssey Infrared Imaging System under the non-saturating conditions. (A–B) Influence of (A) overexpressing and (B) depleting mDpy-30 on the protein level of β1 integrin. The 110 and 130 kDa bands correspond to immature and mature (glycosylated) β1 integrin, respectively. (C) Impact of RbBP5 knockdown on the protein level of β1 integrin. (D) Analysis of β1 integrin mRNA levels with q-PCR in cells depleted of mDpy-30 or RbBP5. Two pairs of primers (Primer set 1: Forward - GCG AGT GTG GTG TCT GTA AG, Reverse - TGT CCC GAC TTT CTA CCT TGG; Primer set 2: Forward - GGA GAA GGA TGT TGA CGA CTG, Reverse - ACA ATT CCA GCA ACC ACA CC) were used to control for possible off-target amplification. Q-PCR was conducted as described in “Materials and Methods”. Variable “n” on each bar graph represents the number of experiments conducted. Error bars: standard deviation.

Given the well established function of the H3K4MT complex in transcription, we then explored whether mDpy-30 and its nuclear interacting proteins modulate the expression of the β1 integrin gene. Indeed, overexpression of mDpy-30 caused the level of β1 integrin protein to decrease (Fig. 8A). On the other hand, compared to a control siRNA, the addition of siRNAs against mDpy-30 correlated with an increase of β1 integrin protein (Fig. 8B). Moreover, suppression of RbBP5 also enhanced the amount of β1 integrin protein (Fig. 8C). Consistent with the increase seen in the protein level of β1 integrin, q-PCR analysis showed that the mRNA level of β1 integrin gene is up-regulated in cells depleted of mDpy-30 or RbBP5 (Fig. 8D). Given that high levels of H3K4 trimethylation are associated with the 5′ regions of almost all active genes [3], we think it is unlikely that the increased mRNA level of β1 integrin is due to decreased H3K4 trimethylation at the promoter region of β1 integrin. Instead, we believe that a factor (or factors) whose expression is sensitive to H3K4 methylation is involved in the transcription of the β1 integrin gene or the regulation (processing or degradation) of β1 integrin mRNA. Regardless of the specific mechanism involved, our collective data shows that mDpy-30 and its interacting proteins regulate the expression of β1 integrin. Since little is known regarding the regulation of β1 integrin expression, our above finding may provide clues on this topic for future studies.

### Effects of abolishing mDpy-30 on cell adhesion after the functional blocking of β1 integrin

**Figure 9:**
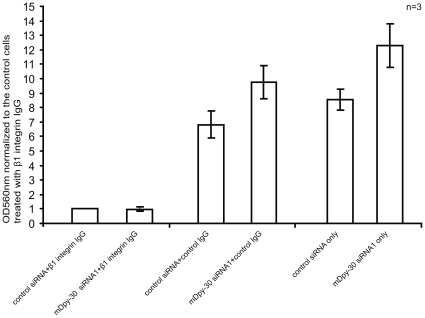
Effects of a functional blocking antibody of β1 integrin on the adhesion of mDpy-30 knockdown cells. The adhesion was carried out as described in “Materials and Methods” except that dissociated U251 cells were first incubated with a blocking anti-β1 integrin antibody (P5D2, mouse IgG1, 10 µg/ml) or a control antibody targeting the nuclear transcription factor AP-2α (5E4, mouse IgG1, 10 µg/ml) at room temperature for 20 min before plating onto a BIOCOAT Collagen I plate. Variable “n” on each bar graph represents the number of experiments performed. Error bars: standard deviation.

The above studies suggest that mDpy-30 and its interacting proteins modulate the subcellular distribution and expression of β1 integrin, as well as cell adhesion/migration. To investigate whether the altered spatial distribution and expression of β1 integrin might contribute to the enhanced adhesion/migration, we assessed the influence of mDpy-30 knockdown on the adhesion in the presence of a functional blocking antibody of β1 integrin [24], [25]. Indeed, blocking of β1 integrin function led to an almost complete loss of the stimulatory impact of mDpy-30 siRNAs on cell adhesion under our experimental conditions (Fig. 9). This data is consistent with the model that mDpy-30 and its interacting proteins regulate cell adhesion/migration, at least partially, via β1 integrin.

Several implications become apparent through our studies. To begin, we have seen that mDpy-30 and its interacting partners can affect cell adhesion/migration, behaviors which are important for numerous physiological and pathological conditions. With this in mind, mDpy-30 and its associated proteins may regulate these processes by controlling the endosomal recycling and expression of key adhesion/migration proteins such as β1 integrin, thus presenting new pharmacological targets. In this regard, recent evidence suggests that CIMPR, the molecule which we have previously shown to be regulated by mDpy-30, might also have a role in cell migration [26]. Second, the observations that knockdown of either BIG1 or RbBP5 affects cell adhesion/migration and β1 integrin targeting, as well as our previous report which shows that knockdown of these two proteins result in the redirection of EGFP-Rab4 and EGFP-Rab11 to cell protrusions [5] suggest that both pools of mDpy-30 are involved. Future experiments are required to determine what roles the nuclear and the Golgi/cytoplasmic pools of mDpy-30 play in the control of the spatial organization of endosomal compartments and thus the endosomal trafficking of CIMPR and β1 integrin. In this regard, it is important to investigate whether mDpy-30 modulates the function of BIG1 or BIG1-associated proteins, and how mDpy-30 and BIG1 coordinate with known regulators involved in endosomal recycling [27]. Additionally, it would also be desirable to identify other trafficking/adhesion/migration molecules whose expression is sensitive to the level of nuclear mDpy-30.

## Materials and Methods

### DNA constructs and reagents

For lentivirus-mediated overexpression of mDpy-30, mDpy-30 cDNA was cloned into pWPI (Addgene). For the EGFP fusions of Rab4b and Rab11, Rab4b and Rab11 cDNAs were cloned into pEGFPC2 (Clontech). The antibodies used in this study are: rabbit anti-BIG2 (Dr. C. Jackson), mouse anti-HA (HA.11, Covance), rabbit anti-HA(ICL), mouse anti-CD8 (Chemicon), rabbit anti-VPS26 (Abcam), mouse anti-CIMPR (BioLegend), mouse anti-β1 integrin (P5D2, Hybridoma Bank), mouse anti-activated β1 integrin (HUTS-21, BD), rabbit anti-Rab4 (Abcam), and rabbit anti-Rab11 (Invitrogen). Alexa 488-conjugated Phalloidin was used to stain F-actin (1∶400 dilution; Molecular Probes). The small interference RNAs (siRNAs) used in this study are: mDpy-30 siRNA1-2 (target sequences, siRNA1: CACCAAATCCCATTGAATT, siRNA2: AAACGCAGGTTGCAGAAAA; QIAGEN), mDpy-30 siRNA3-4 (target sequences, siRNA3: CACAGTTTGAAGATCGAAA, siRNA4: CAGACAACGTTGAGAGAAT; Dharmacon), RbBP5 siRNA1-2 (target sequences, siRNA1:CAGGTGTCTCTCAACAAGCTA, siRNA2: ACGGCAGATCGAATAATCAGA; QIAGEN), BIG1 siRNA1-2 (target sequence: siRNA1: AATGAATCACCTCAACTTAGA, siRNA2: CAGGATTTATTTAGAATTGTT; QIAGEN), Rab4A siRNA (target sequence: AATGCAGGAACTGGCAAA TCT; QIAGEN), Rab4B siRNA (target sequence: CCGCACTATCCTCAACAAGAT; QIAGEN), Rab11A siRNA (target sequence: AAGAGTAATCTCCTGTCTCGA; QIAGEN), Rab11B siRNA (target sequence: CCGCATCGTGTCACAGAAACA; QIAGEN). The non-targeting control siRNAs were obtained from QIAGEN (AllStars Negative Control siRNA) or Dharmacon (On-Target Plus siRNA Control).

### Cell Culture and transfection

HeLa, U251 and U87 cells (ATCC, Rockville, MD, USA) were cultured in Advanced D-MEM medium (GIBCO) supplemented with 4% fetal bovine serum, 2 mM glutamine and 1X penicillin-streptomycin (Cellgro). The same medium was used to grow HeLa cells infected with lentiviral particles and HeLa CD8-CIMPR or stable cell lines except that puromycin (3 µg/ml) and G418 (100 µg/ml) were added, respectively, for maintenance purposes. When needed, cells were transfected with DNA using FuGENE HD (Roche) or siRNA using Lipofectamine RNAiMAX (Invitrogen), and the effects of transfection were investigated 24 (for DNA) or 48 hr (for siRNA) after the transfection, respectively. All siRNAs were used at a concentration of 20 nM.

### Generation and infection of lentiviral particles

HEK293T cells (ATCC, Rockville, MD, USA) were maintained in DMEM medium containing 10% fetal bovine serum and 1X penicillin-streptomycin (Cellgro) until they reached 70% confluence. Two hours before transfection, the medium was replaced with fresh medium preheated at 37°C. For transfection of cells grown on one 15 cm dish, pMD2G (envelop plasmid, 7.9 µg), psPAX2 (packaging plasmid, 14.6 µg), and either pWPI (vector plasmid, 22.5 µg) or pWPI-mDpy-30 (mDpy-30 expressing plasmid, 22.5 µg) were mixed in a 50 ml conical tune, followed by the addition of 0.1X TE (1 mM Tris, 0.1 mM EDTA, pH 8.8, 660 µl), water (350 µl), and 2.5 M calcium chloride (113 µl). After briefly mixing, 1140 µl of 2XHBS (280 mM NaCl, 100 mM Hepes, 1.5 mM Na_2_HPO_4_, 7.11≤pH≤7.13) was added into the mix dropwise under agitation by vortexing to allow precipitation (5 min at room temperature). 2.25 ml of the precipitate was then added dropwise into the cells while mixing gently by rotating the plate. After the medium was replaced with fresh pre-heated medium 14–16 hr post-transfection, supernatant was harvested 3 times every 12 hr and kept at 4°C over the collection period. Finally, the collected supernatants were pooled, centrifuged for 5 min at 1500 rpm, filtered with a 0.22 µm filter, and stored at −80°C in aliquots. To infect cells, U251 were trypsinized, resuspended in the medium containing polybrene (10 µg/ml; Sigma), and allowed to attach to the dish at a confluence of 60–80%. The medium was then removed and kept aside and the virus stock was added with appropriate amount of fresh medium if needed. After letting the virus adsorb for 30–60 min, the medium containing polybrene was added back to the cells. Infected cells were then split and maintained in regular medium until use.

### SDS-PAGE and western blot analysis

Untreated or transfected cells were solubilized in ice-cold RIPA lysis buffer (50 mM Tris-HCl [pH 8.0], 150 mM NaCl, 1 mM EDTA, 1% NP-40, 0.5% sodium deoxycholate and 0.1% SDS, complemented with Complete protease inhibitors (Roche) and 1 mM PMSF, and cleared by centrifugation at 4°C. Cell lysates were quantified using the Non-interfering Protein Assay Kit following the manufacturer's instruction (G-Biosciences) and then denatured in SDS-PAGE loading buffer. An equal amount of total protein per sample was then loaded into each well, separated by SDS-PAGE electrophoresis and transferred to Immobilon 0.45 µM PVDF membranes (Millipore) using a Semi Dry Electroblotting System (Owl). Membranes were incubated with appropriate primary antibody for 1 hr in 1∶1 mixture of Odyssey blocking buffer (Odyssey) and PBS supplemented with 0.1% Tween-20, washed with PBS supplemented with 0.1% Tween-20 (three times for 5 min each), incubated with appropriate secondary antibody (Odyssey) for 30 min in 1∶1 mixture of Odyssey blocking buffer and PBS supplemented with 0.1% Tween-20 and 0.01% SDS, washed again with PBS supplemented with 0.1% Tween-20 (three times for 5 min each) followed with PBS for 5 min, and dried in the dark. Fluorescence quantification was performed on an Odyssey Infrared Imaging System. The same procedure is used for the analysis of histones except that histones were prepared from cells with a Histone Preparation Mini Kit (Active Motif).

### Immunoprecipitation

Transfected cells were solubilized in ice-cold NP-40 lysis buffer (50 mM Tris-HCl [pH 8.0], 150 mM NaCl, 1 mM EDTA, and 1% NP-40, complemented with Complete Protease Inhibitor [Roche] and 1 mM PMSF) and cleared by centrifugation at 4°C. After incubating the supernatant with a primary antibody for overnight followed by protein G-Sepharose (Invitrogen) for 4 hr at 4°C, immunoprecipitates were collected by brief centrifugation. The sepharose beads were washed four times in ice-cold lysis buffer and the bound proteins were eluted with SDS-PAGE sample buffer at 90°C for 10 min.

### *In vitro* binding assay

GST-mDpy-30 was expressed in *E. coli* and purified to homogeneity by glutathione affinity (GSTrap Fast Flow column, GE) followed by size exclusion chromatography (Superdex 200 column, GE). DCB-HUS-Sec7 from human BIG1 (a.a. 2-888) was expressed in baculovirus-infected Sf21 cells as described [15]. The DCB (a.a. 2-224) and Sec7 (a.a. 700-888) domains of human BIG1 were expressed in *E. coli* and purified to homogeneity as previously described [15], [28]. For the pull-down experiments, 100 ml of glutathione Sepharose Fast Flow (GE) was loaded with excess purified GST-mDpy-30 or GST, followed by PBS wash. We optimized our experimental condition to prohibit the BIG1 constructs from binding non-specifically to the empty or GST-loaded column. Purified BIG1 constructs were then added to the mDpy30-loaded column, followed by PBS wash. Bound proteins were eluted with 50 mM glutathione, 50 mM Tris-HCl (pH 8). Eluted proteins were analyzed by SDS-PAGE and Coomassie staining. All experiments were done at least in duplicate.

### GST pull-down

BIG1 fragments were PCR amplified and cloned into pGEX4T2 (Pharmacia). The plasmids were utilized to transform an *E. coli* strain BL21 for expressing the corresponding GST-BIG1 fusion proteins. Purification of fusion proteins was carried out using a GST-Bind Kit(Novagen) and the amount of proteins bound to resins were determined using SDS-PAGE and Coomassie staining after elution. For pull-down, resins were mixed with an equal amount of HeLa crude cell lysates and the amount of mDpy-30 pulled down was assessed using western blot analyses.

### Immunofluorescence

Cells or transfected cells were fixed (in PBS containing 3% formaldehyde, 15 min), permeabilized (in PBS containing 0.1% saponin or Triton, 15 min), blocked (in Blocker Casein in PBS (Pierce) containing 5% goat serum, 20 min), incubated with primary antibody in the blocking buffer (2 hr), washed (in PBS, three times for 5 min each), incubated with fluorophore-conjugated secondary antibody in the blocking buffer (1 hr), washed (in PBS, three times for 5 min each), incubated in PBS containing 1 ug/ml 4′,6-diamidino-2-phenylindole (DAPI, 5 min), and washed (in PBS, three times for 5 min each). Immunostained cells were allowed to air dry, mounted, and examined with Olympus IX-81 microscope.

### Antibody uptake internalization assay

For the kinetics analysis, HeLa-CD8-CIMPR or HeLa cells were blocked and incubated with anti-CD8 or anti-β1 integrin antibody at 0°C to label the surface proteins. After wash, cells were transferred into pre-warmed (37°C) culture medium to allow internalization to proceed for indicated periods of time. For the steady-state analysis, anti-CD8, anti-β1 integrin or anti-CIMPR antibody was added to the culture medium for 45 min at 37°C before fixation to enhance the internalization signal by allowing continuous labeling and internalization. In instances when an acid wash was conducted to remove surface-bound antibody, labeled cells were incubated in ice-cold stripping buffer (0.5 M NaCl, 0.2N acetic acid) for 2–3 mins at 0°C, followed by several washes with ice-cold PBS before fixation.

### Conventional microscopy

An Olympus IX-81 microscope with a PlanApo 60x oil TIRFM objective (1.45 aperture) was used in the epi-fluorescence studies. All microscopy was performed at room temperature. Slides were mounted in *SlowFade* Gold antifade reagent (Invitrogen). Our secondary antibodies were conjugated to either Alexa488 or Rhodamine Red-X (appearing as green and red in the images, respectively). A MediaCybernetics Evolution QEi monochrome digital camera mounted to the Olympus microscope and the InVivo acquisition software version 3.2.0 (MediaCybernetics) were used to capture images and analyze the microscope images. Finally, to crop the images for publication we used Adobe Photoshop and Illustrator software packages.

### Confocal microscopy

Images of the cells were captured with an Olympus Fluoview 500 Laser Scanning Confocal Microscope with a 40X Planapochromat lens, N.A. 1.3. Two laser lines were used, at 488 nm and 543 nm to excite the fluorochromes. Sequential color images capture was used to collect images. To record the distribution of label from apical to basal portions of the cell, z-series were captured of the cells at 0.5 micron steps. Data analysis was performed using Image Pro 6.3.

### ECM adhesion assay

The experiment was conducted on an ECM array (Cell Biolabs) or BIOCOAT cellware (BD) 48-well plates using a colorimetric assay. In brief, cells grown in normal culture medium were dissociated using TrpLE (GIBCO) or Cellstripper (Cellgro), washed once with serum-free Advanced D-MEM medium (GIBCO), re-suspended in the same medium, and finally counted with a hemocytometer. Approximately 100,000 cells were added into each well and allowed to adhere for 45 min at 37°C. After aspirating the medium from each well, cells were washed 5 times with PBS to remove unattached cells and incubated with Cell Stain Solution for 10 min. Following the removal of Cell Stain Solution, cells in each well were again washed 5 times with water and allowed to dry in air. Extraction Solution was then added into each well and the plate was placed on an orbital shaker for 10 min. Finally, Extraction Solution was transferred to a new tube and the OD_560 nm_ was measured.

### Migration assay

The experiment was carried out with a Cell Migration Kit (Cell Biolabs). In brief, cells were dissociated, counted, and re-suspended as described above. Approximately 200,000 cells in serum free medium were added into the top chamber of each well, and serum containing medium was added to the lower chamber. Cells were then incubated at 37°C to allow them to migrate across the 8 µm porous membrane towards the lower chamber. After various periods of time, the serum free medium was aspirated from the inside of the insert and the non-migratory cells on the interior of the insert were removed by wet cotton-tipped swabs. The insert was transferred to a clean well containing Cell Stain Solution for 10 min and washed three times in water. After allowing the insert to air dry, the migratory cells were counted under a microscope. The haptotaxis assay was carried out using a CyotoSelect Cell Haptotaxis Assay kit (Cell Biolabs) with a Collagen I coated membrane. The experiment was performed as described above.

### q-PCR analysis

Total RNA from cells treated with a non-targeting control, or the appropriate siRNA were isolated using an RNeasy Plus Mini Kit (QIAGEN) and quantified with a nanodrop spectrophotometer (Thermo Scientific). cDNA was synthesized by reverse-transcription using the iScript-Select cDNA Synthesis Kit (Bio-Rad) with a nonspecific oligo (dT) primer and qPCR was performed in triplicate for each sample using the iCycler iQ Real-Time PCR System (Bio-Rad). Approximately 100 bp of the cDNA of choice were amplified using two independent sets of primers targeting distinct areas of the mRNA. Fluorescein was included in each reaction to provide a background baseline. Four sets of primers targeting the cDNA sequences of several “house-keeping” gene products were included to determine whether the effects seen were specific to β1 integrin. They are as follows: GAPDH, GPI (3′), HMBS and HPRT1.

## Supporting Information

Figure S1 Characterization of siRNAs against mDpy-30 and RbBP5 in U251 cells. We have previously characterized these siRNAs in HeLa cells using western blot analyses and immunofluorescence assays. To assure the effectiveness of mDpy-30 and RbBP5 siRNAs in U251 cells, we transfected cells with a control non-targeting siRNA (QIAGEN) or each individual siRNA (20 nM, 48 hr) and then examined the knockdown efficiency using western blotting. An equal amount of the total protein was loaded into each lane and GAPDH was used as a loading control.
(0.90 MB EPS)

Figure S2 Characterization of siRNAs against BIG1 in HeLa and U251 cells. HeLa or U251 cells were transfected with a control non-targeting siRNA (QIAGEN) or each of the two different siRNAs against BIG1 (20 nM, 48 hr). The knockdown efficiency of each siRNA was assessed using western blotting or immunofluorescent staining. An equal amount of the total protein was loaded into each lane and GAPDH was used as a loading control. Scale bars: 10 µm.
(8.92 MB EPS)

Figure S3 Characterization of the lentiviral particles expressing mDpy-30. U251 cells were infected with control or mDpy-30-expressing lentiviral particles for 6 days. The lentiviral vector pWPI contains a constitutively expressed GFP allowing the quantification of the infection efficiency; approximately 80% of cells (more than 500 cells scored for each group) remained infected at day 6 when the adhesion/migration assays were carried out. The overexpression efficiency of mDpy-30 was examined using western blotting. An equal amount of the total protein was loaded into each lane and GAPDH was used as a loading control. Scale bars: 10 µm.
(7.61 MB EPS)

Figure S4 Impact of mDpy-30 depletion on the protrusion-targeting of β1 integrin in U251 cells. The β1 integrin endosomal trafficking analysis was performed as described in Fig. 4A. The representative images at 45 min after internalization are shown. Scale bars: 10 µm.
(5.79 MB EPS)

Figure S5 The time course study illustrating the influence of mDpy-30 knockdown on U251 cell adhesion. The adhesion was quantified 20, 40 and 60 min after plating using an ECM array according to the protocol described in “Materials and Methods”. Variable “n” on the bar graph represents the number of experiments performed. Data obtained at 20 min is not shown here due to the inefficient and variable adhesion observed at this time point. Error bars: standard deviation.
(0.92 MB EPS)

Figure S6 The time course study testing the effect of mDpy-30 depletion on the migration of U251 cells. The migration was assessed 2, 4 and 6 hr after seeding using a transwell chamber according to the protocol described in “Materials and Methods”. Variable “n” on the bar graph represents the number of microscope fields examined. Error bars: standard deviation.
(0.47 MB EPS)
